# The genetic signature left by the range expansion of red foxes in Australia is detectable after more than 80 years of population stability

**DOI:** 10.1002/ece3.11212

**Published:** 2024-04-04

**Authors:** Stephen D. Sarre, Aaron T. Adamack, Yvette Hitchen, Carl D. Soulsbury, Bernd Gruber, Oliver F. Berry

**Affiliations:** ^1^ Centre for Conservation Ecology and Genomics, Institute for Applied Ecology University of Canberra Canberra Australian Capital Territory Australia; ^2^ Science Branch, Northwest Atlantic Fisheries Centre Fisheries and Oceans Canada St. John's Newfoundland and Labrador Canada; ^3^ School of Biological Sciences The University of Western Australia Crawley Western Australia Australia; ^4^ Helix Molecular Solutions Leederville Western Australia Australia; ^5^ School of Life & Environmental Sciences University of Lincoln Lincoln UK; ^6^ National Collections and Marine Infrastructure, CSIRO, Indian Ocean Marine Research Centre The University of Western Australia Crawley Western Australia Australia

**Keywords:** allele surfing, biological invasions, founder effects, rapid expansion, recapitulation

## Abstract

Reconstructing biological invasions from historical sources can provide insights into how they occur but are difficult to do when invasions are poorly documented. Genetic signatures left by invaders can also offer insights into invasion routes, points of origin and general biology but often present conclusions that are contradictory to expectations. Here, we test the ability of continental‐wide microsatellite genotype data from 29 loci and 3122 samples to reconstruct the well‐documented invasion of red foxes *Vulpes vulpes* from the United Kingdom into Australia over 150 years ago, an invasion that has led to the extinction of many native species. Our analysis reveals several key signals of invasion evident in Australian foxes. They display lower levels of diversity than foxes sampled from the UK, exhibit clines in diversity from the point of introduction (south‐east Australia) to the edge of their range, and show strong evidence of allele surfing in westerly and north‐easterly directions. These characteristics are consistent with a single point of origin followed by rapid expansion in westerly and north‐easterly directions as suggested by historical records. We also find little genetic structure in foxes across Australia with only the vast Nullarbor Plains and Great Victoria Desert region presenting a detectable barrier to their dispersal. As such, no mainland region within the current range of foxes can be considered genetically isolated and therefore appropriate for localised eradication efforts. Overall, our analyses demonstrate the ability of comprehensive population genetic studies to reconstruct invasion histories even after more than 80 years since colonisation was stabilised.

## Graphical abstract

We use the well‐documented invasion of red foxes into Australia to advance understanding of the genetic signature left by wildlife when introduced into new territory. We found that while Australian foxes exhibit little genetic sub‐structuring they do display lower levels of genetic diversity, clines in diversity from the point of introduction to the edge of their range and strong evidence of allele surfing in westerly and north‐easterly directions. These characteristics are consistent with their well‐documented introduction at a single point of origin followed by rapid expansion and demonstrate the ability of comprehensive population genetic studies to accurately reconstruct invasion histories even when there is little evidence of population genetic structure.

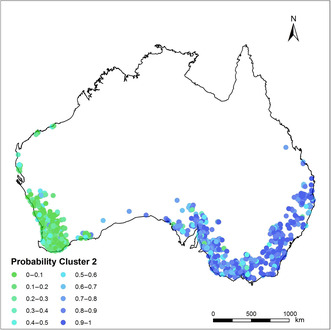

## 1 INTRODUCTION

Understanding the origins, dynamics and course of biological invasions can provide clues to the management and eradication of the invaders and the prevention of future incursions (Banerjee et al., 2022; King, 2023; Konečný et al., 2013; Lee, 2002). Yet, reconstructing those processes from historical sources, sometimes well after the invasion has occurred, can be difficult and will generally give only part of the story. Population genetic analyses can provide useful information about such invasions because a species expanding its range undergoes a series of founder events (Slatkin & Excoffier, 2012) leaving a genetic signature (Excoffier et al., 2009; Hamelin & Roe, 2020; Slatkin & Excoffier, 2012) that can provide information about invasion routes and points of origin and the strengths and weaknesses in the biology of the invading species (Brazier et al., 2022; Short & Petren, 2011). Nevertheless, verification of these expectations and the theory underlying the tests for them (Excoffier et al., 2005) is difficult given the timescales required for such effects to occur, the rate at which migrants are exchanged per generation (Excoffier et al., 2009) and a paucity of population genomic studies (Matheson & McGaughran, 2022). Added complexity arises from land‐use modification, the introduction of new species, climate change and multiple other pressures affecting species distributions (Moran & Alexander, 2014; Stuart et al., 2022; Van Der Putten, 2012) which combine to reduce the ability of genetic markers to distinguish among ecological phenomena. For example, declines in heterozygosity and allelic richness and increases in genetic differentiation, phenomena that theory predicts should occur towards the edge of expansion ranges, are not observed consistently in nature (Swaegers et al., 2013). Similarly, tests for genetic bottlenecks often fail to detect even severe population declines (Charbonnel et al., 2022; Peery et al., 2012). In this context, examples that draw on historical references while applying comprehensive genetic analyses provide the opportunity to test the veracity of available approaches and identify what is likely or unlikely to be provided by them.

When the range of a species expands, allele frequencies often form a gradient between the population core and its new range limits as only a proportion of the alleles present in the core area survive and disperse (surf) the expanding populations. This process can cause dramatic shifts in allele frequency that would not occur in a stable population (Chuang & Peterson, 2016; Miller et al., 2020) and may involve allelic sweeps without the conferral of any selective advantage (Hallatschek et al., 2007). Overall, repeated founder events and genetic surfing should decrease heterozygosity and allelic diversity as distance from the core (ancestral) population increases (Austerlitz et al., 1997; DeGiorgio et al., 2011) while some alleles, even those that are rare in the core, may reach high frequencies at the edge of the range (Edmonds et al., 2004; Slatkin & Excoffier, 2012). These effects should leave a genetic signature of greater genetic differentiation among populations near the range limits than those at the core (Nei et al., 1975).

The detectability of a range expansion is weakened by the number of generations since the expansion began, the number of individuals sampled, the residual effects of past range expansions and contractions, the demographics of the species and an incomplete knowledge about the ways in which a species uses particular landscapes (Garroway et al., 2011; Gracia et al., 2013). One empirical way of testing genetic detection for recent range expansions is to contrast the genetic signals of invasive species for which an invasion history is well documented with the genetic signals expected given its invasion history. This approach minimises the effects of many of the artefacts of historical changes in range and population size that may affect the strength of a signal of range expansion. By combining the results of tests for range expansion for a species known to have undergone one, it may be more possible to identify the conditions under which the various genetic signals of range expansions are likely to be detectable. By doing this, we can better apply these genetic signals to other species with undocumented histories.

**FIGURE 1:**
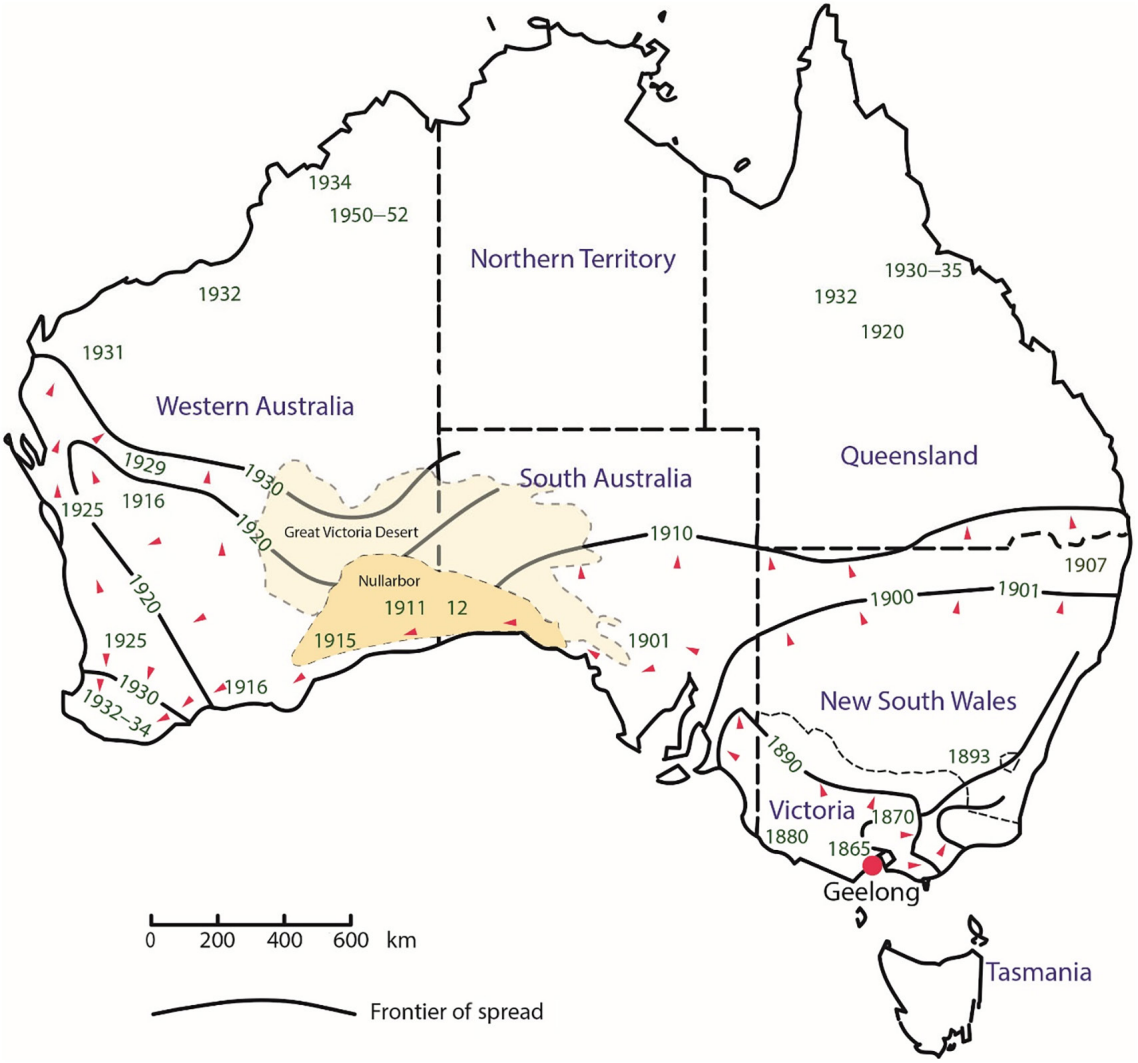
Frontier of spread for the red fox in Australia from the release point in Geelong circa 1865. Red arrows indicate the direction of spread. After Saunders et al. (1995) Managing Vertebrate Pests: Foxes. Bureau of Rural Sciences, Australia.

The red fox, *Vulpes vulpes*, provides one candidate species for testing for the genetic consequences of range and demographic expansions following its introduction into Australia. Red foxes are ecologically plastic, found in a wide range of ecosystems from deserts, boreal forest to urban areas (Elmhagen et al., 2017; Fleming et al., 2021; Lovell et al., 2022) and have long‐distance dispersive abilities (Colson et al., 2017; Walton et al., 2018). The ~150‐year invasion history of red foxes in Australia has been well documented (Jarman, 1986; Rolls, 1969) through newspaper extracts and other sources (Abbott, 2008, 2011). Foxes were repeatedly brought from the United Kingdom to an area near Geelong in the state of Victoria, Australia (Figure 1) for the purpose of hunting and somewhere between the high 10s to low 100s were released into the wild on at least five occasions between 1845 and 1873 (Abbott, 2011). Once foxes became established in the Werribee district of Victoria between 1874 and 1878 they spread rapidly westward across Australia at ~20 km/year, and north‐eastward into New South Wales at ~30 km/year (Abbott, 2011; Fairfax, 2019; Figure 1) reaching the Adelaide district in 1905 and the east coast of Western Australia by around 1924 having crossed the Nullarbor Plain into Western Australia at a rate of 400 km/year or more (Abbott, 2008). Foxes now number more than 7.2 million (McLeod, 2004), representing an incredibly rapid colonisation of around 76% (~5.79 million km^2^; Figure S1) of the continent (Jarman, 1986; West, 2008) with profound implications for Australian biodiversity including multiple extinctions (Kinnear et al., 2002; Saunders et al., 2010; Stobo‐Wilson et al., 2022; Woinarski et al., 2015).

Red foxes in Australia therefore offer a unique opportunity to examine key genetic signatures during a biological invasion. First, their well‐documented introduction history makes clear that a founder event is likely to have occurred. Second, the small number of founding animals transported from the UK to Australia will have created a genetic bottleneck. This scenario is supported by evidence from mitochondrial DNA in which only two haplotypes, both of which are from the same subclade as most British and Irish red foxes, have been identified (Statham et al., 2014) implying a narrow bottleneck. From theory, we expect decreases in genetic diversity, heterozygosity, allele number and size range in Australian foxes when compared to those of the UK. Third, foxes in Australia have undergone rapid westward and north‐eastern range expansions over thousands of km from their point of origin near Geelong, Victoria. We therefore predict that heterozygosity and allelic richness will decline with increasing distance from Geelong in both directions (Austerlitz et al., 1997; DeGiorgio et al., 2011).

Here, we present a comprehensive, continental‐wide survey of microsatellite DNA loci of Australian foxes to test for these genetic signals. Specifically, we test for gradients in allele frequency between the core population (as defined by the reported point of introduction) and those at the range limits, decreases in genetic diversity with distance from the core, and higher levels of genetic structure at the range edges relative to the range core. Our results reveal that some, but not all, genetic expansion signals of foxes are detectable on a continental scale even after almost a century of stable distribution and suggest that genetic surveys post‐expansion can reveal key characteristics about a species invasion for many generations.

## 2 MATERIALS AND METHODS

### 2.1 Sampling

Australian fox samples were obtained through a media campaign that encouraged public participants to collect tissue samples from road‐killed animals or from foxes that were killed as part of existing culling programs between April 2006 and April 2008. Collected samples were preserved in lysis buffer (Longmire et al., 1997). Participants provided locations for all samples, either as latitude and longitude, property identifiers, addresses or with reference to distance from landmarks. Sample collection was approved by the University of Western Australia Animal Ethics Committee. UK fox tissues (*N*) were collected from around Britain (England, Scotland and Wales; Table S1) for a separate project (Edwards et al., 2012) and sub‐sampled for this current project.

### 2.2 Laboratory analysis

DNA extraction and microsatellite DNA genotyping were conducted using the methods described in Berry and Kirkwood (2010). Thirty‐six microsatellite DNA loci, consisting of loci derived from the dog (*Canis familiaris*) and red foxes were genotyped along with the sex (SRY) marker. In brief, 10 multiplexed PCR reactions were required, enabling fragment analysis of all markers in 2 capillary lanes. We conducted 10 μL PCR reactions consisting of 5 μL of Qiagen PCR Multiplex Supermix (Qiagen, Hilden, Germany), 2 μL of DNA, primers and ultrapure water using PCR conditions as described in Berry and Kirkwood (2010). Genotypes were checked by eye in the GENEMARKER software (Softgenetics LLC) and 96 samples were genotyped twice to determine error rates.

### 2.3 Data analysis

Quality Control: We performed a number of quality control steps on the Australian fox genotypes, including tests for the presence of null alleles and departures from Hardy–Weinberg equilibrium (Adamack & Gruber, 2014) and linkage disequilibrium (GENEPOP 4.2; Rousset, 2008). We also ran analyses in R (Team, 2018) to determine the fraction of loci that were retained following quality control and successful genotyping and the fraction of individuals that were successfully genotyped at each locus. Furthermore, as our dataset spans the Australian continent (approximately 4000 km from east to west), it is likely that the assumption of random mating that underpins tests for Hardy Weinberg equilibrium and linkage disequilibrium would be violated. To reduce the risk of this occurring, we subdivided our dataset spatially into 1° latitude by 1° longitude grid cells and tested for HWE and linkage disequilibrium by grid cell. This process led to the exclusion of 7 loci (Ren.94K, Ren01G01, Ren186, FH2793, FH3767, WanV402, C10.602) owing to a combination of linkage disequilibrium, poor genotyping success and/or frequent departures from Hardy–Weinberg equilibrium, leaving 29 loci for our final analyses all of which appear to be in equilibrium across single grid cells. Individual animals were also excluded from the analysis if the number of loci successfully genotyped was <60%. Following these quality control measures, we retained 3122 individuals for Australia and 50 individuals for the UK for the complete analysis. A smaller number of foxes were used for portions of the analysis described below (Section 2.3).

### 2.4 Testing for fox population structure

We tested for genetic structure among Australian foxes using STRUCTURE 2.3.4 (Falush et al., 2003; Pritchard et al., 2000). We performed two main sets of runs (one with the LOCPRIOR option and one without) in STRUCTURE. For both, we assumed an admixture ancestry model with correlated allele frequencies and ran STRUCTURE for *K* equal to 1–10, with 20 replicates being run for each level of *K* with each replicate incorporating 10^5^ burn‐in steps followed by 2 × 10^5^ MCMC steps. The optimal number of clusters was determined following Evanno et al. (2005) and as implemented by STRUCTURE HARVESTER (Earl & vonHoldt, 2012). To confirm the optimal number of clusters as determined by STRUCTURE HARVESTER, we used CLUMPP (Jakobsson & Rosenberg, 2007) to align cluster labels across replicate runs for *K* = 2 to optimal *K* and then compared barplots of cluster assignments across replicates using R to ensure that assignments were consistent across runs and that there were no ‘ghost clusters’ (Durand et al., 2009). Once we had identified the main population clusters, we repeated the analysis for each cluster to uncover any evidence of sub‐structuring should it exist. This process was repeated until no further sub‐clusters were identified.

### 2.5 Testing for patterns in genetic diversity

**FIGURE 2:**
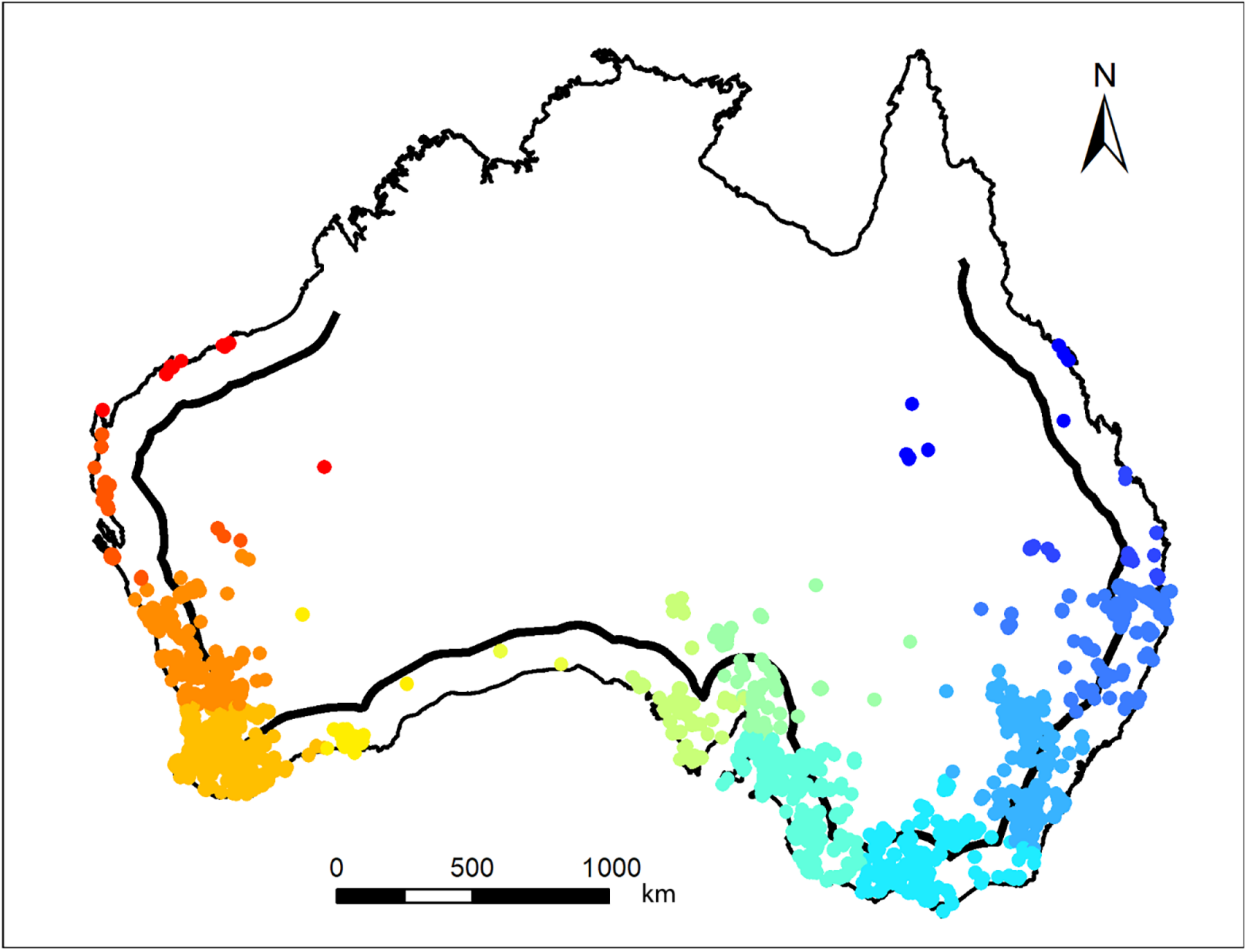
Sites of tissues collected from Australian foxes 2006 to 2008. The thick black line marks the 150 km line inland from the coast around the interior of Australia used to signify the approximate path along which foxes spread following their release at Geelong. Circle colours denote the equidistant groupings identified through mapping samples to the 150 km line but where groups with less than 10 individuals were merged with adjacent groups to produce the 14 groups depicted.

To facilitate genetic analysis we divided the Australian fox population into sub‐groups, by converting the two‐dimensional distribution of Australian foxes into a one‐dimensional (linear) distribution. This was accomplished by transforming fox positions onto a line 150 km inland from the coast around the interior of Australia. Foxes that were located more than 150 km away from the nearest point on the line were dropped from the analysis leaving 2679 foxes. This line followed the approximate path along which foxes spread following their release at Geelong, Victoria to Western Australia and up the east coast of Australia towards Queensland (Figure 1; Jarman, 1986). That line was then partitioned into 18 equidistance segments which we treated as population groups. Groups with fewer than 10 individuals were merged with adjacent groups resulting in a total of 14 groups with *N* ranging from 10 to 789 animals (Figure 2). For each group of individuals, we determined their median distance from Geelong along the line. We tested for Hardy–Weinberg equilibrium and linkage disequilibrium within each of these 14 groups using PopGenReport (Adamack & Gruber, 2014) and GENEPOP 4.2 (Rousset, 2008) respectively. All multiple comparison tests were conducted using a strict Bonferroni correction set to a family‐wide significance of *p* < .05.

We determined allele frequencies at each locus for each of the 14 groups and calculated the sum of the rarefied allelic richness across loci, the expected heterozygosity for all loci and the median distance of each group from Geelong. Mantel tests were performed comparing distance matrices for a group's median path distance to Geelong to distance matrices for the frequency of each allele, the sum of the allelic richness across loci and the expected heterozygosity. Separate tests were performed for foxes moving west, from Geelong to Western Australia and for foxes moving east and north, from Geelong to Queensland. We used a strict Bonferroni correction (*α* = .05/number of alleles/locus) to adjust for the large number of tests performed to examine changes in allele frequency.

We also examined trends in allele frequencies moving west and northeast from the core area. Specifically, we calculated the frequency for each allele in each group across all 29 loci and performed Mantel tests (with and without Bonferroni corrections) to test for decreases in genetic diversity while also calculating the mean allelic richness with rarefication and (mean) heterozygosity across all retained Australian and UK foxes as well as each Australian spatial group. While binning the distances from the origin partly removes the correlation between distances, this will not be the case for all distance pairs. As such, using the Mantel test helps to eliminate biases arising from differences in correlations between distances. We visualised these data using plots of allelic richness and mean heterozygosity comparing UK to Australian foxes and allelic richness (sum of alleles and median number of alleles per loci) and mean heterozygosity to distance from Geelong (for both western and north‐eastern expansion routes). We performed separate Mantel tests for the western and north‐eastern expansion routes and tested for increases in genetic differentiation at the range edges by plotting Nei's GST between all spatial groups and the average of the pairwise Nei's GST between adjacent spatial groups against distance from Geelong.

Finally, we examined gene flow independently of grid cell groups through the implementation of EEMS (Estimating Effective Migration Surfaces; Petkova et al., 2016) which enables the visualisation and quantification of spatial patterns of genetic differentiation across geographic regions. EEMS estimates effective migration rates over a continuous landscape by modelling genetic dissimilarity between individuals or populations in relation to their geographic locations. The method operates by creating a Voronoi tessellation over the study area, where each cell in the tessellation represents a different migration rate. The rates of migration are inferred from the genetic data, under the assumption that areas with higher rates of gene flow (migration) will have lower genetic differentiation. EEMS then uses a Bayesian framework to infer these rates, integrating over uncertainty in the migration surface. This approach allows for the visualisation of genetic structure in a way that highlights barriers to gene flow and regions of high connectivity, providing insights into the historical and current patterns of movement and gene flow across the landscape.

## 3 RESULTS

### 3.1 Genotyping

A total of 3279 Australian fox samples were returned between April 2006 and April 2008. Samples came from much of the fox's range across the Australian continent, but the vast majority were collected within ~300 km of the coast and within the 300 mm rainfall isohyet (Anon, 2017) where fox densities are highest and human activity is high. From those 3279 samples, we were able to extract genetic information from 3122 individuals with the required quality (Berry et al., 2023). For all analyses, where it was necessary to allocate individuals into groups along the coastline, we restricted our analysis to animals <300 km from the coastline because attribution of individuals further away than 300 km would not be possible without some arbitrary decision as to which group individuals would belong.

### 3.2 Allelic diversity and heterozygosity

We found a diminished number and distribution of alleles in Australian foxes compared with those sampled from the UK (Table 1) consistent with that expected between a population founded by a small number of individuals and its source. Specifically, we identified fewer alleles (404 cf 459) among all Australian foxes sampled (*n* = 3122) compared with the 50 foxes sampled from across England, Wales and Scotland while the mean number of alleles per locus was almost half that found in the UK (7.01 cf 13.26). Mean heterozygosity among loci was also lower in Australian foxes (0.71 cf 0.79) than those in the UK while Australian foxes contained 53 fewer private alleles (151 cf 206). While it is possible that these comparisons are skewed by reductions in genetic variation towards the edges of their range in Australia, the general lack of genetic structure in Australian foxes suggests that this is unlikely to be a significant influence on these results.

**TABLE 1:** Comparison of microsatellite DNA genotypes from Australian and UK fox samples.

	Australia	UK
Number of individuals	3122	50
Total number of alleles	404	459
Number of private alleles	151	206
Mean no. alleles/locus	7.01	13.26
Mean expected heterozygosity	0.71	0.79

### 3.3 Genetic structure

**FIGURE 3:**
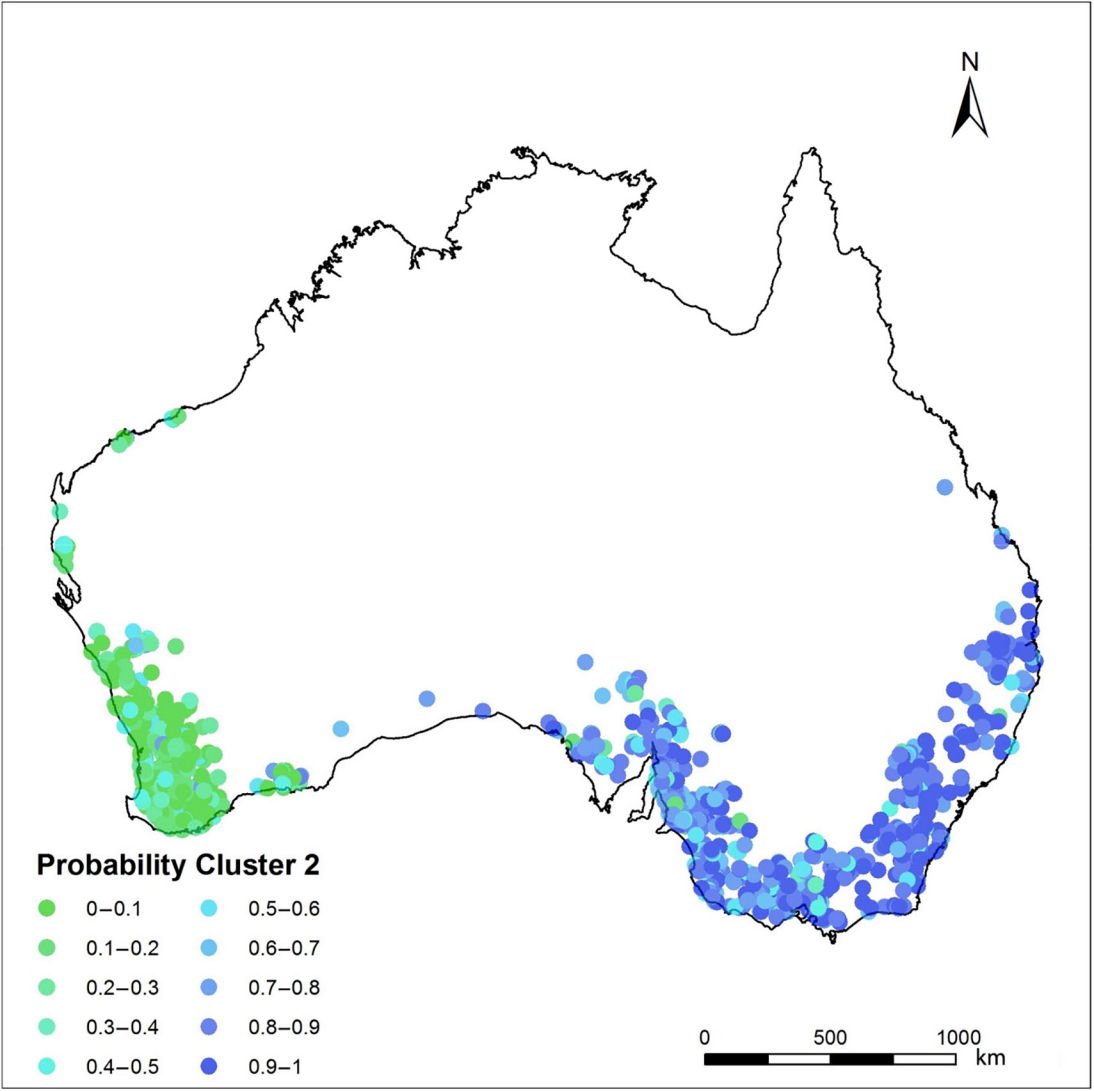
Cluster assignment for each sample using sampling locations (LOCPRIOR) as a prior assumption for cluster assignments. STRUCTURE HARVESTER indicated that a structure of *K* = 2, is consistent across replicates. Each dot represents a genotype with green and blue dots corresponding to western and eastern genetic clusters respectively.

**FIGURE 4:**
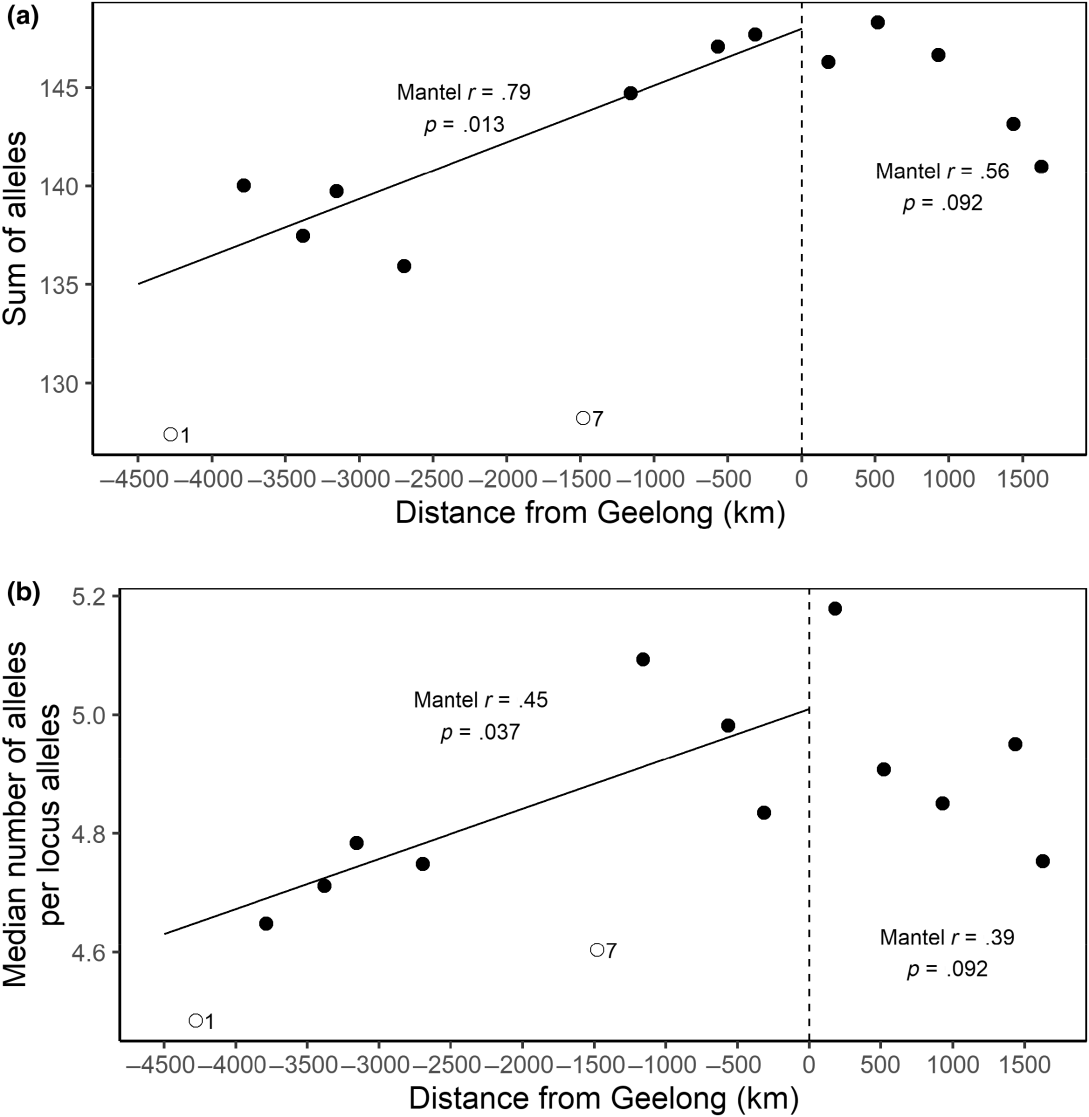
Trends in (a) sum and, (b) median, number of alleles per locus by sub‐group. Sub‐groups to the west of Geelong have negative distances while sub‐groups to the east of Geelong have positive distances. Trendlines are shown when trends were found to be significant using Mantel tests. Open circles represent outlier sub‐groups. We consider the low values of these sub‐groups as likely to be a function of their small sample sizes (one and seven animals respectively) and have excluded them from the Mantel tests.

**FIGURE 5:**
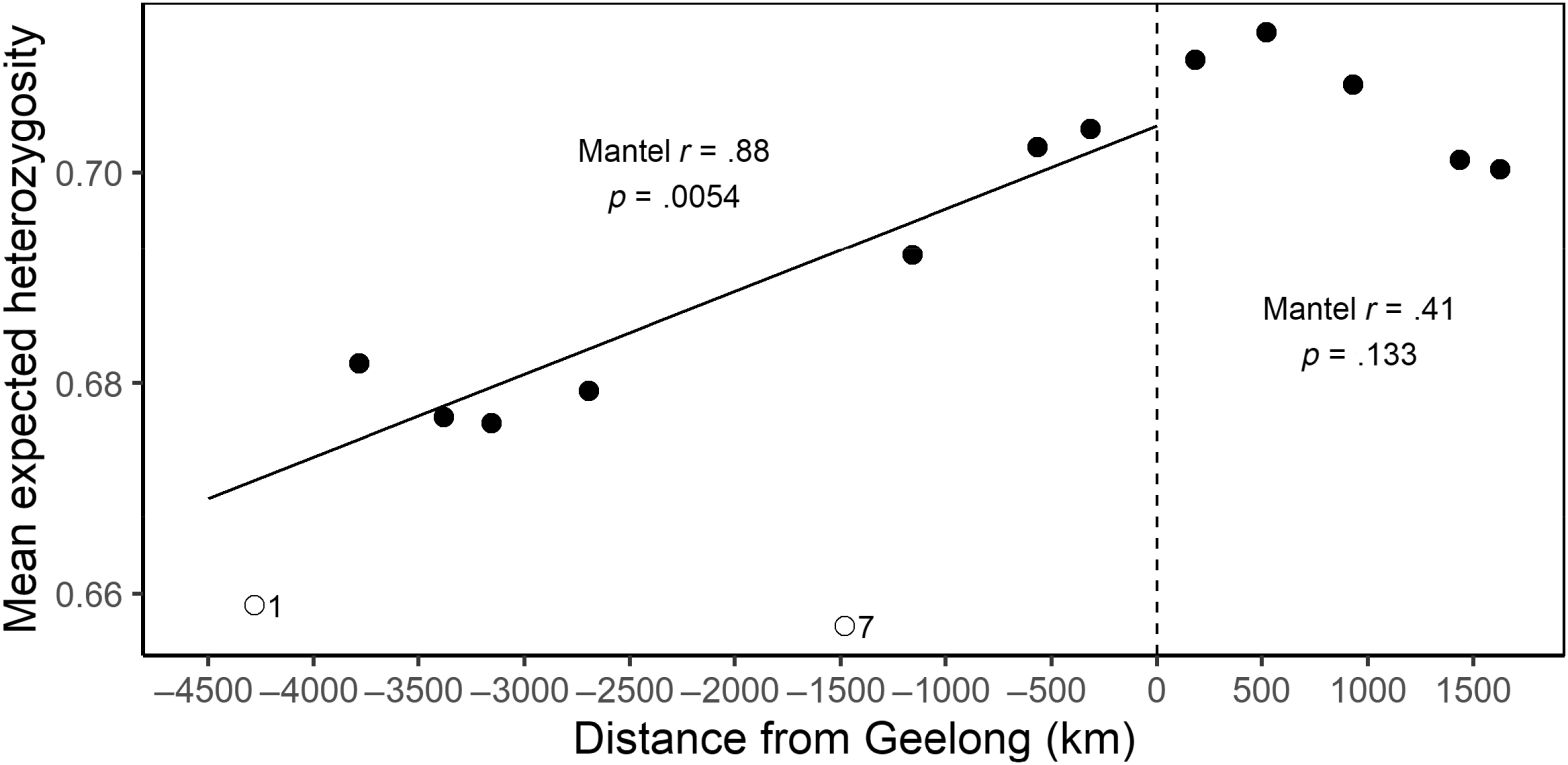
The mean expected heterozygosity by sub‐group as a function of the sub‐group's distance (km) from Geelong, Victoria. Sub‐groups to the west of the point of release (Geelong) have negative distances while sub‐groups to the east have positive distances. Trendlines are shown when trends were found to be significant using Mantel tests. Open circles represent two outlier sub‐groups with very low levels of mean heterozygosity. We consider the low values of these sub‐groups as likely to be a function of their small sample sizes (1 and 7 animals, respectively) and have excluded them from the Mantel test.

**FIGURE 6:**
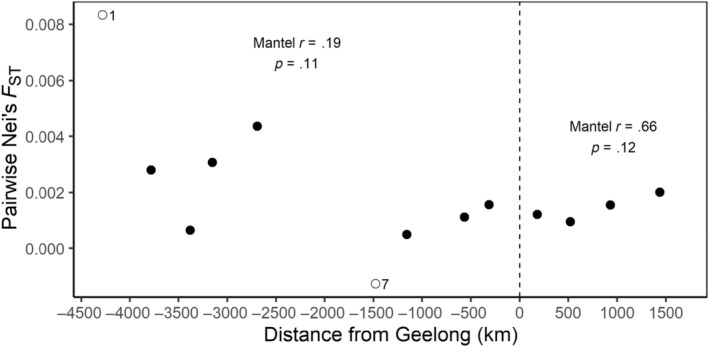
Pairwise *F*_ST_ by sub‐group as a function of the sub‐group's distance (km) from Geelong, Victoria. Sub‐groups to the west of Geelong have negative distances while sub‐groups to the east of Geelong have positive distance.

STRUCTURE analysis revealed that the genetic structure of Australian foxes was best described by a subdivision into two clusters (*K* = 2) with one cluster, west of the Nullarbor Plain (a large, mainly treeless plain about the size of New Zealand) containing all individuals sampled from the Western Australian end of the population and all other foxes east of the Nullarbor Plain constituting the second. Subsequent analyses failed to identify any further sub‐clustering within either main cluster (Figure 3). We found strong evidence of clines in allelic frequency with distance from the point of release (Geelong) in the western expansion with 45 (14) of 308 alleles showing this trend (*p* < .05 (*p* < .05 with Bonferroni correction)) and to a lesser extent in the eastern expansion (15 (1 with Bonferroni correction) out of 353 alleles). Declines away from the point of release were evident in the westward expansion with significant (*p* < .05) declines in both the total number of alleles with rarefication (*r* = .68 and *p* = .0024) and the median numbers of alleles per locus (*r* = .34; *p* = .034) with fewer alleles at the western margins than at Geelong (Figure 4a,b). Declines in allelic richness were not significant for the eastward expansion (sum of alleles with rarefication: *r* = .56 and *p* = .092; median number of alleles per locus: *r* = .39 and *p* = .092) and in mean heterozygosity (west: *r* = .87; *p* = .005; east: *r* = .77, *p* = .05; Figure 5). In contrast, pairwise *F*_ST_ values did not change significantly with distance from the point of release (Figure 6) although there appeared to be a general trend of increasing *F*_ST_ with distance in both directions. We found strong evidence of allele surfing at some loci (Table 2), where individual alleles that exhibited higher or lower frequencies in line with a cline pattern, but were not disproportionally higher or lower. Overall, genetic diversity was lower at the edge of the expansion than at its core. This gradient is more evident going east to west than south to northeast. The EEMS analysis (Figure S2) also identifies the Nullarbor as a key barrier to gene flow and higher migration rates along the coasts but suggests that there may also be restricted gene flow between the east coast foxes and those in central southern Australia.

**TABLE 2:** Proportion of alleles with significant trends away from the point of origin (Geelong) in Australian red foxes.

	Western Australia	Eastern Australia
Number of alleles examined	380	353
Number of alleles with insufficient data to determine the trend	94	104
Number of alleles with a significant trend (after Bonferroni correction)	45 (14)	15 (1)
Proportion of alleles with significant trends (after Bonferroni correction)	0.12 (0.037)	0.042 (0.0028)

*Note*: Clines in allele frequencies are indicative of allele surfing towards the edge of the range.

## 4 DISCUSSION

Our analysis of microsatellite DNA variation in Australian foxes recapitulates closely the genetic effects expected from its well‐documented introduction into Australia in the 19th Century and its subsequent rapid expansion (Jarman, 1986; Rolls, 1969; Saunders et al., 2010). The signature of their introduction and expansion is clearly visible through reduced genetic diversity (relative to the UK source population) and continental clines in the distribution of genetic variation that align with the proposition, based on historical records (Abbott, 2011; Jarman, 1986), that there was a small number (10s to low 100s) of individuals introduced at a single location. As such, it suggests that comprehensive genetic surveys can pinpoint sites of introduction and directions of expansion where there is little or no information enabling contemporary reconstructions of invasion histories.

### 4.1 Reductions in genetic diversity in Australian foxes

We show that consistent with founder theory, the Australian fox population exhibits substantially lower levels of diversity (allelic richness, heterozygosity, numbers of private alleles) than foxes sampled from the United Kingdom with lower numbers of private alleles seen in the Australian population. By comparison, mean expected heterozygosity and allelic richness are not significantly lower in the Australian population although they are reduced. These differences are consistent with the elimination of rare alleles through a short population bottleneck and the retention of heterozygosity through a subsequent rapid expansion. The reductions in levels of genetic variation of Australian foxes compared with those of the parent population clearly have not been an impediment to the success of the fox as a colonising species as it established a continental‐wide distribution, covering over 5600 km (linear distance) within around 70 years of release.

### 4.2 Single point of origin for Australian foxes

The patterns of genetic variation at the continental scale are strongly suggestive of a single point of origin for Australian foxes. Decreases in allele number and heterozygosity with distance from Geelong and strong evidence of allele surfing at the western and northern edges of the range all point to an area near Geelong as the point of introduction. These trends are most evident in the western expansion. Combined, they indicate a single point of origin commensurate with the reported release site near Geelong followed by rapid movement westward and a less marked northeast expansion. This difference between the strength of the patterns exhibited may arise simply as a difference in scale (>4000 km heading west cf ~1500 km northeast) or it may reflect the different porosities of the western and eastern regions to foxes. In particular, the western expansion crosses the Nullarbor Plain and the Great Victoria Desert which sit across the border between South Australia and Western Australia (Figure 1) whereas there are no such expanses encountered by the northern‐eastern expansion. The Nullarbor is a sparsely inhabited arid and semi‐arid region of over 548,000 sq km with a maximum width of about 1100 km east to west. While not impenetrable it is a sufficiently significant barrier that far‐western foxes form a cluster that is distinct from those in the east. The gap in sampling observed across the Nullarbor area is likely to be caused by a combination of low fox densities and poor sampling given its remote location and small human population (Figure S1). We note that sightings of foxes as they expanded west suggest that it took only around 15 years from (1901 to 1915/16) to expand across the Nullarbor and Great Victorian Desert regions (Saunders et al., 1995). In contrast, the north‐eastern expansion was through largely arable land with higher rainfall and more productive soils and likely represented a more benign environment to the dispersing foxes.

The clines in heterozygosity, allelic richness and the sum of alleles observed moving from the east to the western seaboard, could be formed in part by the apparent restriction in gene flow across the desert regions but it is difficult to be definitive about this given the scarcity of fox samples from the Nullarbor/Victoria Desert. Conversely, we could find no influence of the other significant topographical feature on the Australian mainland—the Great Dividing Range. This range, which varies in width from about 160 km to over 300 km, runs the entire length of the eastern coastline stretching from north‐eastern Queensland to the Grampians in western Victoria has no clear influence on fox population genetic structure even with an unusually large sample of microsatellite loci. We conclude that this range does not present a significant barrier to fox dispersal although that permeability is almost certainly facilitated by roads and bridges. A more intensive sampling is required to test this proposition.

### 4.3 Implications for the fox invasion of Australia

There are few estimates of fox dispersal distances in arid Australia but our finding that genetic clusters are detectable only at the continent level suggests that dispersal distances for this species are large and that only the Nullarbor Plain and Victoria Desert provide a barrier sufficient to inhibit dispersal. Even then, our data indicate that gene flow occurs between the two clusters despite the vastness of those arid regions and demonstrates that fox control for nature conservation or agricultural production purposes must operate on a very large scale to avoid rapid reinvasion. Data from more regional studies showing little evidence of genetic structure support these observations (Watson et al., 2021) and are consistent with the low levels of genetic structuring seen in modern and ancient European foxes (Teacher et al., 2011). With the exception of island populations (Berry & Kirkwood, 2010) no region within the current range of foxes can be considered isolated and therefore appropriate for localised eradication. So, unless fox control for conservation occurs within arid regions where fox population recovery or recolonization can be slow (Berry et al., 2013), dispersal may negate the benefits of localised fox control, even when the areas of control are large, by preventing the suppression of foxes for sufficient time to enable affected prey populations to recover.

Our analysis of introduced foxes into Australia reveals much about the way they achieved such an astonishingly quick colonisation of a vast and largely arid continent that is environmentally very different from their population of origin in the United Kingdom. That expansion took around 70 years to extend across extremely inhospitable arid lands but mirrors, and may even have profited from, the single introduction of wild rabbits to an area near Geelong approximately 10 years before foxes (Alves et al., 2022). The signal among foxes is stronger in the western expansion than in the northeast even though the latter covered a distance of over 1500 km from the point of release. Foxes reached the NSW/Queensland border at about 1905 only some 19 years ahead of their arrival near the west coast of Western Australia suggesting that the difference in genetic signature is not particularly one of timing, but more likely one of distance covered or habitat heterogeneity that has made for the rapid homogenisation of neutral genetic variation in foxes along the east coast of Australia. There is little doubt that the Australian East Coast holds conditions for foxes that support population higher densities than those in the arid areas (Saunders et al., 1995) to the west of Victoria. The resolution provided by genotype‐by‐sequencing approaches may be required to detect expansions when gene flow is high and the population expansion substantial.

## 5 CONCLUSIONS

The distribution of genetic variation seen in Australian foxes recapitulates the known history of the fox expansion since their initial release in the 1860s. It shows a single likely point of origin that aligns closely to the recorded release history and demonstrates the characteristics expected of a bidirectionally expanding population over 70 years since that expansion was complete. The ability to detect these key features of an invading predator suggests that comprehensive genetic surveys such as the one presented here can enable the pinpointing of points of introduction and the direction of expansion in species for which there is little or no information about their release. In so doing, it presents an approach for contemporary reconstructions of invasion histories for focal species that may enable points of entry and the direction of invasion to be determined. Further analyses, utilising whole genome genotyping, will provide the capacity to identify key genomic elements of invasive species across the type of allelic gradients that we have observed, providing clues about the genetic elements that promoted the initial invasion and which may present opportunities for future control.

## AUTHOR CONTRIBUTIONS

**Stephen D. Sarre:** Conceptualization (supporting); formal analysis (supporting); supervision (supporting); writing – original draft (lead); writing – review and editing (lead). **Aaron T. Adamack:** Data curation (supporting); formal analysis (equal); visualization (equal); writing – review and editing (equal). **Yvette Hitchen:** Data curation (equal); formal analysis (equal); methodology (equal). **Carl D. Soulsbury:** Investigation (supporting); resources (supporting); writing – review and editing (equal). **Bernd Gruber:** Data curation (supporting); formal analysis (equal); writing – original draft (supporting); writing – review and editing (supporting). **Oliver F. Berry:** Conceptualization (equal); data curation (lead); formal analysis (supporting); funding acquisition (lead); investigation (lead); methodology (equal); project administration (equal); supervision (lead); visualization (supporting); writing – original draft (supporting); writing – review and editing (supporting).

## CONFLICT OF INTEREST STATEMENT

The authors declare no conflict of interests.

## BENEFITS GENERATED

A research collaboration was developed between scientists from Australia and the UK providing genetic samples and conducting the analyses. Sample collection, involving a citizen science‐based approach, built awareness among the broader scientific and lay communities. All collaborators are included as co‐authors and the results of the research have been shared with the provider communities and the broader scientific community through the Invasive Animals Cooperative Research Centre (now the Centre for Invasive Species Solutions – https://invasives.com.au/).

## Supporting information


Appendix S1.

## Data Availability

Individual genotype data and their metadata are available at https://doi.org/10.5281/zenodo.10020047.
