# Supplementary material for: The genetic signature left by the range expansion of red foxes in Australia is detectable after more than 80 years of population stability
Source: Ecol Evol. 2024 Apr 4;14(4):e11212. doi: 10.1002/ece3.11212 (PMC10994981; doi:10.1002/ece3.11212)
Supplement: Supplementary file 1 — Appendix S1. [file ECE3-14-e11212-s001.docx]

Table S1: Locations of UK foxes genotyped for the

present study. Data from C. Soulsbury unpublished.

| Collection No | Location |
| --- | --- |
| 1 | Bristol, UK |
| 2 | Bristol, UK |
| 3 | Bristol, UK |
| 4 | Bristol, UK |
| 5 | Bristol, UK |
| 126 | Scotland, UK |
| 824 | Scotland, UK |
| 129 | South England, UK |
| 117 | Central England, UK |
| 113 | Central England, UK |
| 121 | Scotland, UK |
| 127 | Scotland, UK |
| 132 | Northern England, UK |
| 298 | Central England, UK |
| 131 | Wales, UK |
| 660 | South-western England, UK |
| 627 | South-east England, UK |
| 659 | South-east England, UK |
| 686 | Wales, UK |
| 685 | Central England, UK |
| 28 | Northern England, UK |
| 813 | South-east England, UK |
| 267 | Central England, UK |
| 280 | South-east England, UK |
| 547 | East England, UK |
| 56 | East England, UK |
| 224 | Not provided |


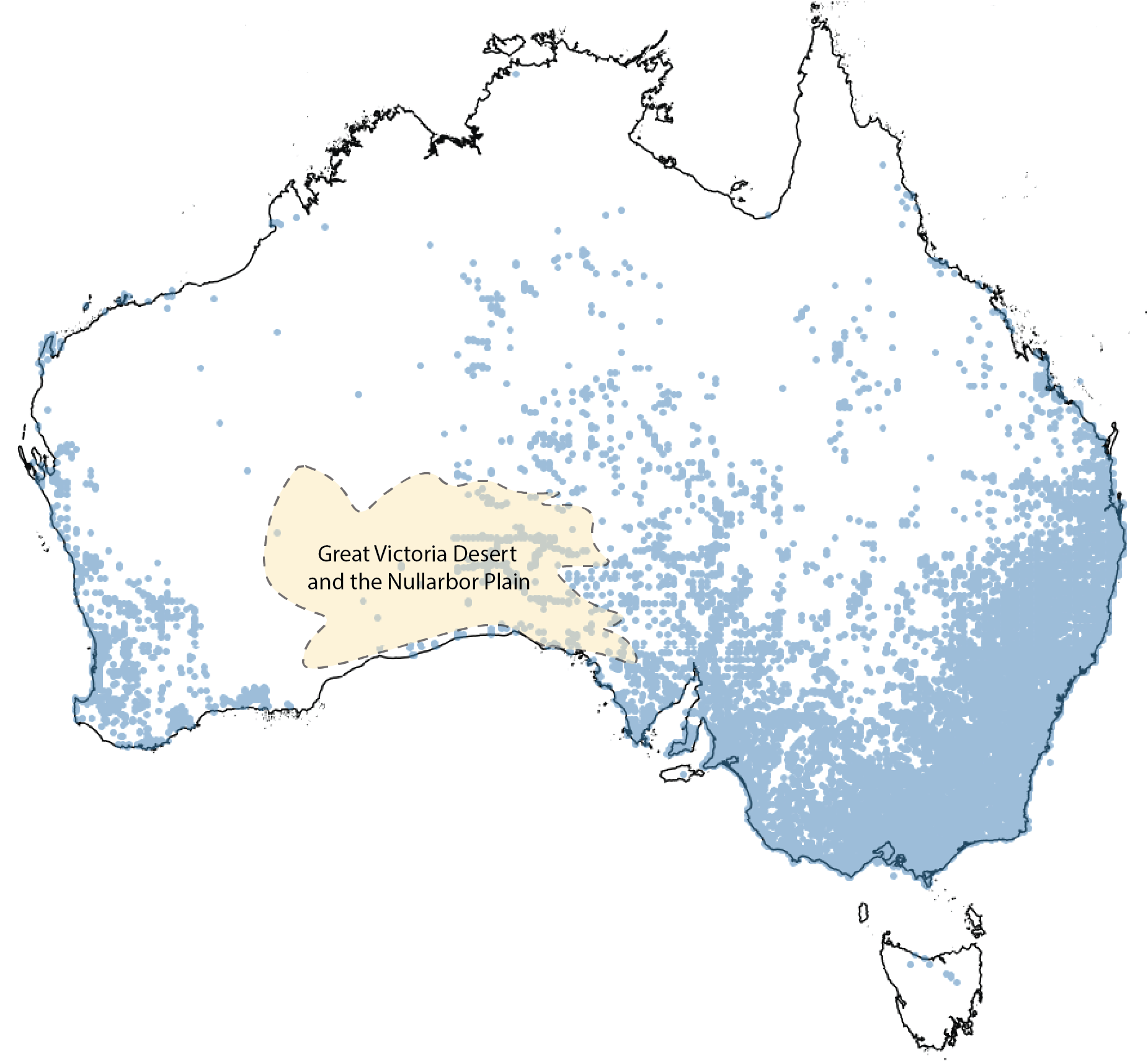


**Figure S1**. Reported fox observations in Australia 1865 to present. Each dot represents a single observation. Data from the Atlas of Living Australia using the search criterion “*Vulpes vulpes*”. Downloaded September 25, 2023. *https://biocache.ala.org.au/occurrence/search?q=raw_taxon_name%3A%22Vulpes+vulpes%22#tab_mapView*.

**
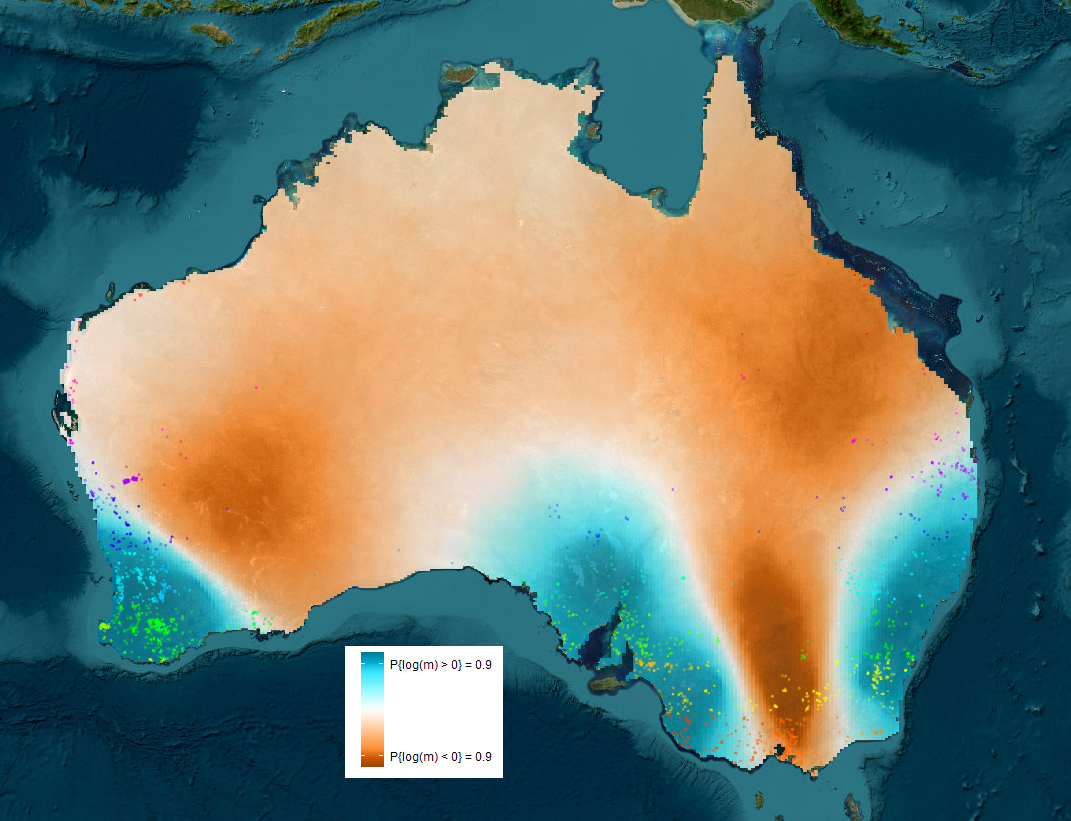
**

**Figure S2**. Colour contour plot for Australian foxes produced by averaging draws from the posterior distribution of the migration rates, interpolating between grid points and using genomic data from 3122 foxes collected across their range and produced following the approach of Petkova et al. (2016); log(m) denotes the effective migration rate on a log10 scale, relative to the overall migration rate across the habitat.
